# StOPping Hypertension and imprOving Children’s Lives after KidnEy TranSplantation (SOPHOCLES): study protocol for a randomized controlled multicenter trial

**DOI:** 10.1186/s13063-025-09033-z

**Published:** 2025-08-27

**Authors:** Carl Grabitz, Nima Memaran, Rizky I. Sugianto, Jeanine von der Born, Mila Bukova, Elena Lehmann, Ann-Kathrin Konuhov, Dennis Holzwart, Anika Großhennig, Elke Wühl, Bernhard M. W. Schmidt, Anette Melk

**Affiliations:** 1 Department of Pediatric Kidney, Liver, Metabolic and Neurological Diseases, Hannover Medical School, Hannover, Germany; 2 Interdisciplinary Transplantation Research Group, Hannover Medical School, Hannover, Germany; 3 Division of Pediatric Nephrology and Gastroenterology, Department of Pediatrics and Adolescent Medicine, Comprehensive Center for Pediatrics, Medical University of Vienna, Vienna, Austria; 4 Institute of Biostatistics, Hannover Medical School, Hannover, Germany; 5 Division of Pediatric Nephrology, Center for Pediatrics and Adolescent Medicine, Heidelberg University Hospital, Heidelberg, Germany; 6 Department of Nephrology and Hypertension, Hannover Medical School, Hannover, Germany; 7 Hannover Medical School, Children’s Hospital, Carl-Neuberg-Str. 1, Hannover, 30625 Germany

**Keywords:** Kidney transplantation, Arterial hypertension, Left ventricular hypertrophy, Telemonitoring, Cardiovascular disease, Pulse wave velocity, Intima media thickness, Graft function

## Abstract

**Background:**

Cardiovascular disease is a major morbidity in children after kidney transplantation, limiting life expectancy and impairing graft function. Arterial hypertension is the dominant cardiovascular risk factor and highly abundant in this patient group. Arterial hypertension can cause left ventricular hypertrophy, which is predictive of cardiovascular death. Left ventricular hypertrophy can be non-invasively assessed by measuring left ventricular mass. Observational data indicated that intensified blood pressure control was associated with a significant reduction of left ventricular mass. Based on evidence from the randomized controlled ESCAPE trial, intensified blood pressure control is recommended in children with chronic kidney disease prior to kidney replacement therapy. However, current treatment recommendations for pediatric kidney transplant patients follow the recommendations for otherwise healthy children and adolescents with arterial hypertension, i.e., suggesting a blood pressure target < 90th percentile.

**Methods:**

In the proposed multicenter, randomized, parallel group trial with blinded endpoint evaluation, we aim to include 170 pediatric patients who underwent a kidney transplantation more than 12 months ago. Patients will be randomly assigned 1:1 to an intensified blood pressure management group (blood pressure target ≤ 60th percentile) and a standard blood pressure management group (blood pressure target < 90th percentile). The primary endpoint is left ventricular mass after 24 months. Secondary endpoints are pulse wave velocity, intima media thickness, estimated glomerular filtration rate, and albuminuria. Achievement of blood pressure targets will be facilitated through blood pressure telemonitoring. Blood pressure values will be transmitted in real time to the treating physician and the trial’s centralized study office allowing timely responses in case blood pressure values lie outside target range.

**Discussion:**

The proposed study will result in an evidence-based definition of blood pressure targets and will therefore have direct implications for the care of children after kidney transplantation. In case intensified blood pressure targets are effective, this should eventually lead to lower cardiovascular morbidity and subsequently lower cardiovascular mortality of pediatric kidney transplant recipients.

**Trial registration:**

ClinicalTrials.gov NCT06589947. Registered on September 6, 2024.

**Supplementary Information:**

The online version contains supplementary material available at 10.1186/s13063-025-09033-z.

## Background

Life expectancy in children with chronic kidney disease (CKD) after kidney transplantation (KTx) has improved steadily [1]. Therefore, factors affecting long-term health, especially cardiovascular (CV) disease, carry increased relevance. CV morbidity is caused by a variety of risk factors. Of those, arterial hypertension (AH) is of particular importance, as it is strongly correlated with CV organ damage [2, 3]. CV events are among the most common causes of death in pediatric KTx recipients [4–6]. Studies demonstrated a high prevalence of AH in pediatric KTx recipients [7, 8] with more than 70% requiring antihypertensive medication [9] and more than one third showing actual blood pressure (BP) levels higher than the 95th percentile (pct) [8]. A longitudinal analysis from the Cooperative European Pediatric Renal Transplant Initiative (CERTAIN) Registry of 336 pediatric KTx recipients showed a prevalence of AH of 84% at discharge after KTx that declined only slightly to 77% after 3 years of follow-up [8]. We extended this finding, demonstrating sharply increased odds for AH for patients after pediatric KTx when compared to matched healthy children in a case–control study [10]. Long-term data report AH in more than 60% of pediatric KTx recipients after 10 years [11]. Several studies demonstrated the negative impact of AH on CV health and transplant survival in children after KTx [12, 13].

Left ventricular hypertrophy (LVH) is the most prominent manifestation of CV organ damage as a result of AH [14]. LVH is associated with death due to major CV events [15] and affects about 40% of children 1 year after KTx [16, 17]. We have previously shown that the left ventricular mass index (LVMI), an echocardiographic parameter to quantify LVH, increases in the early course after pre-emptive KTx and is mainly determined by BP and not by the mode of kidney replacement therapy (KRT) [18]. LVH is a strong predictor of CV morbidity and mortality [19–22]; therefore, measurement of left ventricular mass is a cornerstone of CV risk assessment [14, 23].

In children with CKD not yet requiring KRT, the randomized controlled ESCAPE trial demonstrated that intensified BP control using angiotensin-converting-enzyme (ACE) inhibition reduces CKD progression [24]. This pivotal study has led to the implementation of BP goals for patients prior to KRT. However, currently no specific recommendations or guidelines exist on BP management in pediatric patients after KTx that are based on prospective data. To discern the effect of different BP levels and thereby the importance of BP control on LVH in KTx patients, we analyzed long-term follow-up data from the 4C-T study [25], a sub-study of the 4C study [26], which prospectively assessed CV morbidity in children prior to and after KTx. We analyzed LVMI depending on BP control in 94 pediatric KTx recipients. The cumulative systolic/diastolic BP exposure was calculated as time-averaged area under the curve and categorized according to pct ranges (≤ 50th, > 50th– ≤ 75th, > 75th– ≤ 90th, > 90th pct). We showed that a cumulative exposure to systolic BP values ≤ 75th pct is associated with lower LVMI: Compared to patients with a cumulative systolic BP exposure > 90th pct, a significant LVMI reduction of − 5.24 g/m^2.16^ was seen in patients exposed to systolic BP between 50th and ≤ 75th pct (*p* = 0.007). A similar tendency was observed in those exposed to ≤ 50th pct (*ß* = − 3.70 g/m^2.16^; *p* = 0.067), while no LVMI reduction was found in patients with cumulative systolic BP exposure between 75th and ≤ 90th pct. The cumulative exposure to lower diastolic BP levels was also associated with lower LVMI with no large differences in the magnitude of the effect on LVMI across the three strata. Patients exposed to systolic BP ≤ 50th or between 50th and ≤ 75th pct had a significant risk reduction of 79% or 83% lower odds for the development of LVH, respectively. A similar tendency was seen for diastolic BP with patients exposed ≤ 50th pct showing 82% lower odds for LVH [25].

These findings substantiate that continuous exposure to high BP levels leads to LVH progression in pediatric KTx recipients, but also clearly point out that the conservative BP goal < 90th pct is most likely insufficient to lower LVMI, especially for systolic BP. This further supports the hypothesis that a lower BP goal will be beneficial with regard to LVH, leading to a reduction in CV disease and improved long-term patient survival. However, as these observations are inferred from observational data only, a randomized controlled trial (RCT) prospectively evaluating the effect of different BP goals on LVMI (primary endpoint) is warranted. In fact, the KDIGO (Kidney Disease: Improving Global Outcomes) guideline on BP in patients with CKD recommends performing an RCT on the adequate BP goal in children, ideally using home- or office-based BP measurements [27].

Measurements for subclinical vascular damage as well as for graft function will comprise our secondary endpoints. The surrogate markers for vascular damage will be aortic pulse wave velocity (PWV) and carotid intima media thickness (IMT). Both have been shown to be predictive of CV mortality in adults [28, 29] and are recommended parameters to be used as CV endpoints in studies by the American Heart organization [30]. We have demonstrated in a variety of both single-center and multicenter observational studies that both PWV and IMT can be non-invasively and reproducibly assessed in a variety of pediatric cohorts [17, 31–34]. We will assess the estimated glomerular filtration rate (eGFR) and albuminuria as measures of graft function and glomerular integrity, as, again both can be easily measured.

## Methods/design

### Aim of the study

The SOPHOCLES trial targets a central clinical problem highly relevant for the studied patient population and addresses a major knowledge gap. The proposed trial aims to prove whether intensified treatment of AH (i.e., BP control ≤ 60th pct, compared to standard BP control < 90th pct) leads to a lower LVMI in KTx recipients. Reducing LVMI will reduce long-term morbidity and improve quality of life and life expectancy in this population particularly vulnerable to CV disease. The results have therefore great potential to change future clinical practice.

### Outcome parameters

SOPHOCLES’ primary endpoint is LVMI at 24 months after randomization. In children and young adults after KTx, the incidence of CV events (e.g., myocardial infarction or stroke), despite being 100-fold higher compared to the normal population, is still too low to provide adequate power for an RCT with reasonable duration. LVH has not only been shown to predict the incidence of CV events and CV mortality [19], but more importantly, a decrease in left ventricular mass is associated with a decrease of CV risk [35–38]. This makes LVMI a unique endpoint that ideally reflects CV risk [14]. Furthermore, LVH assessment is also recommended in the European guidelines for pediatric hypertension [23]. Therefore, LVMI is the most suitable endpoint for this trial.

Secondary endpoints will be PWV as measure of arteriosclerosis, IMT as measure of atherosclerosis, eGFR as a measure of graft function, and albuminuria as a surrogate for glomerular integrity.

### Design of the trial

**Fig. 1:**
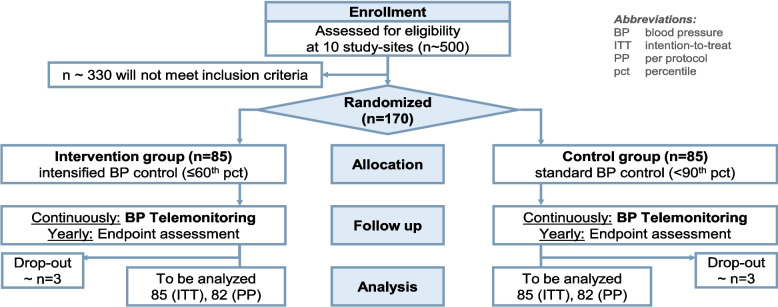
Patient flow diagram

The trial is a multicenter, randomized, controlled, parallel group trial with blinded endpoint evaluation (see Measures against bias below) comparing the effect of intensified vs. standard BP control on left ventricular mass for superiority. The flow diagram (Fig. 1) illustrates the trial design and depicts the patient numbers from enrollment to analysis.

### Study population

Inclusion criteria: We seek to include patients between 6 and 18 years, who had undergone KTx > 12 months ago and are diagnosed with AH.

Exclusion criteria: Patients with cardiac malformation cannot be included. We will also exclude patients, who have undergone treatment for a rejection episode within 3 months prior to inclusion, or who actively participate in a randomized double blinded trial of a new investigational drug within 30 days prior to inclusion.

### Patient recruitment

The participating centers provide care to a large number of pediatric KTx recipients allowing to screen more than 500 patients for eligibility. As highlighted in the trial’s flow chart (Fig. 1), we expect patients to be excluded because of age or not suffering from AH. Past experience taught us that pediatric KTx patients have a close relationship to their treating physicians and show a high willingness to participate in clinical studies. We expect to recruit the necessary number of patients within 24 months. Participating centers are academic hospitals within Europe and contributors to the CERTAIN registry [8]. The majority of patients will be recruited in Germany. An up-to-date list of centers will be maintained at ClinicalTrials.gov (NCT06589947).

### Informed consent

Each family will receive detailed information on the purpose and procedures of the trial. In addition to thorough verbal information, a patient information form (provided in German and languages spoken by patients and their families) adapted to the respective age of the child will be handed out, and any questions arising will be answered. This includes information of potential risks and benefits. Signature will be given by the legal guardians and if possible by the child itself. If participants reach the age of 18 years during the course of the study, they will be asked to re-consent at the following study visit. No trial specific tasks will be performed before reaching informed consent. The signed informed consent forms will be filed by the local investigators.

The study will be conducted in accordance with the Declaration of Helsinki in its current version. Participation is completely voluntary. Consent can be withdrawn at any time, without obligation to provide justification and without affecting future medical care. In the event of withdrawal from the study, any data or material obtained thus far will be requested to be used in context of the study purpose. Otherwise, data and biomaterial will be destroyed, unless already analyzed.

### Study procedures

This trial will compare two different BP treatment goals. Pediatric KTx recipients will be randomly assigned to the intensified BP group or the standard BP group. Treatment goal in the intensified group will be lowering BP ≤ 60th pct. Data justifying this treatment goal are discussed in the introduction. The treatment goal for the standard group is to achieve BP levels < 90th pct in accordance with pediatric guidelines for AH [23, 39]. The chosen antihypertensive drug regimen is in the discretion of the treating physician.

All patients will measure BP at home using a telemonitoring device (Tel-O-Graph, IEM GmbH, Aachen, Germany) providing current BP data and allowing for the patients’ accustomed mode of documentation. The device enables telemonitoring through real-time transmission of BP data, which will be accessible to the MHH trial physician at the trial’s centralized study office and to the treating physician on-site allowing for timely interventions and as an important safety feature.

Physicians caring for the patients will have no restrictions in treating medical conditions (e.g., intercurrent infections or rejections) or changing any medication as part of the regular clinical care. There are no prohibited concomitant medications in the context of this trial. Changes in medication and any adverse events (AE) or serious adverse events (SAE) will be recorded in the electronic case report form (eCRF).

### Investigational plan, assessment of endpoints

**Fig. 2:**
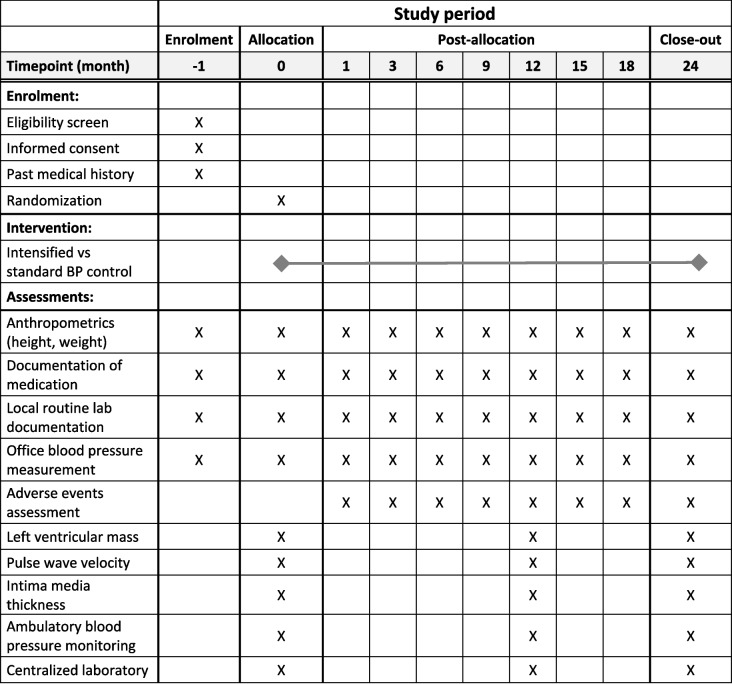
Schedule of enrollment, intervention, and assessments

Figure 2 provides the timepoints and scope of the planned trial visits.

Echocardiography will follow a standardized operating procedure established during the 4C study in accordance with the American Society of Echocardiography guidelines [40]. LVMI will be calculated by dividing left ventricular mass in grams (according to Devereux et al. [41]) by height in meters to the 2.16th with a correction factor of 0.09 [42]. LVH is defined as an LVMI ≥ 45 g/m^2.16^ independent of sex or age. PWV will be provided from oscillometric measurements using the Vicorder device (Skidmore Medical Ltd, Bristol, UK) following established standardized operating procedures [3]. IMT will be assessed using ultrasound with a high-resolution linear probe, by averaging five measurements from the far wall of the common carotid artery 1–2 cm proximal of the carotid bulb, following the Mannheim consensus [43]. Appropriate normative data allow for PWV and IMT to be normalized by age and sex and to be expressed as *z*-scores. Blood and urine samples will be analyzed. eGFR will be calculated using the modified pediatric Schwartz formula [44]; albuminuria will be measured in spot urine and provided as albumin-to-creatinine ratio.

### Study timeline, study duration

**Fig. 3:**
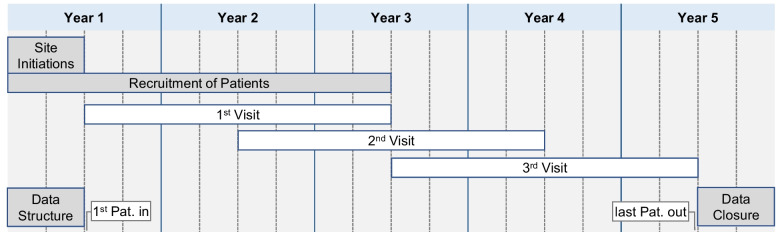
Study timeline

Trial duration will be 24 months for each patient (Fig. 3). This time span is sufficient to reach the BP goal and to induce changes in LVMI. The stringent BP monitoring allows for a fast adaption of antihypertensive treatment with the aim to reach the target within 3 months. A shorter trial duration is not advisable as LVMI changes need time [45]. We expect the first visible changes to occur no sooner than after 6 months in the intensified BP group. As the magnitude of these changes increases over time, a longer trial duration allows for the recruitment of a smaller number of patients. Our analysis of 4C-T data [25] demonstrated a robust difference in LVMI dependent of BP control after a median follow-up of 26 months. Hence, we based the trial duration on these data.

Pediatric patients > 12 months after KTx are routinely seen every 4–6 weeks in the outpatient clinic. The proposed quarterly trial visits (Fig. 2) can therefore be easily implemented without additional strain on patients. The annual evaluation will take approximately 45 min per patient. The non-invasive measurements include evaluation of LVMI by echocardiography, ambulatory blood pressure monitoring, and assessment of secondary endpoints that will be executed by the same investigator team. Moreover, collected biomaterial will be analyzed centrally to guarantee standardization of laboratory values.

The duration of the whole study will be 5 years.

### Adherence/rate of loss to follow-up

From our experience from previous studies including RCTs, we expect an excellent follow-up adherence resulting in a low number of drop-outs [24, 46]. This is facilitated as all pediatric KTx recipients are under close, continuous surveillance at the participating centers, generally seen every 4–6 weeks. Thereby, the additional burden through the study is very low. The number of visits to the outpatient clinic essentially remains unchanged; only during the annual visits patients and their families will have to take extra time for the CV examinations. Echocardiography and the other CV examinations are non-invasive and known to be well-tolerated. Blood samples will be collected in context of a routine blood draw. The trial’s design using telemonitoring approach will also facilitate very good protocol adherence.

### Sample size calculation

Our published data from the 4C-T study [25] was the basis for sample size calculation. To simulate the SOPHOCLES trial as best as possible, we performed an additional analysis on a subgroup of 37 patients very closely resembling the inclusion and exclusion criteria for the planned trial. At baseline, patients were 6–16 years old, were transplanted ≥ 9 months ago, and presented with AH. We observed the lowest LVMI corrected mean value in patients who were exposed to cumulative systolic BP of ≤ 75th pct (47.4 ± 12.0 g/m^2.16^) when compared to patients exposed to values > 90th pct (52.3 ± 11.5 g/m^2.16^) or patients between > 75th and ≤ 90th pct (52.7 ± 11.4 g/m^2.16^). We considered the observed difference of 5.3 g/m^2.16^ in LVMI achieved between the ≤ 75th pct and the > 75th– ≤ 90th pct as clinically relevant. A closer look at patients below ≤ 75th pct showed a skewed distribution with a median systolic BP exposure of 57.5th pct (IQR: 51st–66th pct, maximum: 71st pct) supporting our proposed BP target of ≤ 60th pct.

A sample size calculation based on the corrected means of the subgroup analysis (47.4 vs. 52.7 g/m^2.16^), a common standard deviation of 12 g/m^2.16^, an unpaired *t*-test with a two-sided type 1 error of 5% and a power of 80% resulted in a sample size of at least 82 patients for each group (nQuery® 9.2.1.0.). We expected only few drop-outs and therefore only added 3 patients per group to account for withdrawal of consent. This resulted in an overall sample size of 170 patients. Importantly, it was assumed that the planned mixed model approach in the primary analysis will increase power compared to the *t*-test used for sample size calculation because of stratification, highlighting our conservative approach in setting up this trial.

### Measures against bias

Permuted block randomization with variable block length will be used to allocate patients 1:1 to both groups. The following strata will be used: study center, sex (male or female), age group (< 11 or ≥ 11 years), and LVH (< 45 g/m^2.16^ vs. ≥ 45 g/m^2.16^). The randomization sequence will be generated by a dedicated team from the Institute of Biostatistics overlooking the entire randomization process. Randomization is performed using web-based randomization tool (secuTrial) and executed by the central study office.

All echocardiographic and secondary endpoint readings will be performed by the same investigators who will be uniformly trained according to the standards established as part of the 4C study [26]. The analyses of these readings will be performed centrally by investigators blinded with regard to the patient’s intervention group and time point of the measurement.

As a result of an increased awareness for BP control by treating physicians on site, we expect an improvement in BP levels also in the standard group during the trial. The telemonitoring approach, however, will assure clear differentiation between both groups. The transmission of BP data and the close contact between MHH trial physician and treating physicians will assure strict target control and immediate corrections, if targets are not met. For generalizability, we aim to recruit a representative patient population. In consequence, the only exclusion criterion is cardiac abnormalities that very rarely occur, but would impede measurement of the primary endpoint.

### Primary analysis

According to the intention-to-treat (ITT) principle, analysis of the primary endpoint will include all randomized patients. A linear mixed model for repeated measures (MMRM) will be used to analyze the contrast in LVMI (least squares means at month 24 after randomization) between groups. Treatment group, LVMI at baseline, visit, the stratification factors (center, sex, age group, presence of LVH), and treatment-by-visit interaction will be included as fixed effects. Before analysis, the patient numbers in the centers will be assessed in a blind review. Small centers (with less than 20 patients in the center) may be consolidated for analysis using a pendulum strategy (i.e., combining the largest with the smallest center, the second largest with the second smallest center). Patient will be included as random effect; an unstructured covariance pattern is assumed to model the within-patient errors. Restricted maximum likelihood in combination with the Newton–Raphson algorithm will be used to obtain parameter estimates. The Kenward-Roger method will be used for estimation of the denominator degrees of freedom. Superiority of intensified treatment will be concluded, if the upper boundary of the 95% confidence interval for the estimated treatment effect (contrast of intensified minus control group at month 24) is below 0. In the unlikely situation that the model does not converge, a simplified model (i.e., simplified covariance pattern, such as compound symmetry, or a fixed effects model) will be used for the primary analysis. Missing values will be implicitly imputed by the MMRM.

To assess the robustness of the model, various sensitivity analyses will be performed. Specifically, the impact of missing values (e.g., replacement by last-observation-carried-forward in an ANCOVA model) will be examined. Nature and extent of missing values will be compared between treatment groups and a per-protocol analysis, which consists of all patients who complete the study in accordance to the study protocol, will be conducted. Furthermore, subgroup analyses will be performed for age group, sex, and center.

### Secondary analysis

Analysis of secondary endpoints (PWV, IMT, eGFR, and albuminuria at month 24 after randomization) will be performed in line with the primary analysis.

AEs and SAEs will be documented and recorded. We will calculate relative and absolute frequencies of AEs per intervention arm at the event level and at the patient level. A two-sided chi-squared test will be used to compare the intervention and control group. Expected AE related to the study intervention can occur due to lowering of BP per se (dizziness, nausea, weakness, but also acute worsening of kidney function) and due to side effects of the antihypertensive medications (e.g., edema from calcium antagonists, cough from ACE inhibitors, electrolyte disorders from diuretics).

### Stopping rules

On an individual level, the patients receive no specific treatment but are assigned to different treatment goals. In this setting, we believe that any stopping rules carry the risk of inducing bias into our study. Patients’ safety is guaranteed by telemonitoring of BP values allowing for timely reaction if BP levels are too low. This situation then will prompt adaption of treatment but not exclusion from the study. The only reason for early stopping of the study is withdrawal of patients’ consent, unforeseeable new ethical or medical aspects. A single center might wish to stop the study due to logistic reasons.

In context of the whole study the primary endpoint is left ventricular mass measured after 2 years. The recruitment period also spans 2 years (Fig. 3). Hence, a meaningful preliminary endpoint assessment of, e.g., half of the patients would only be possible very late in the trial. Therefore, stopping the trial at this time point, e.g., due to an overwhelming positive result, would not be reasonable.

### Risk–benefit assessment

The principal study aim is ethically justified because of the scientific uncertainty which of the two interventions (intensified vs. standard BP management) will result in a more favorable risk–benefit profile. While observational data suggest that stricter BP control will translate into a reduction of left ventricular mass and better patient survival, the intensified protocol might have disadvantages as well. These may be due to worsening of overall treatment adherence because of an increased drug burden, but also due to potential side effects of lowering BP (such as dizziness or even acute worsening of graft function), or side effects coming from antihypertensive medication itself (e.g., edema from calcium antagonists, cough from ACE inhibitors, electrolyte disorders from diuretics). In conclusion, we believe that clinical equipoise is assured.

Both groups will benefit from improved monitoring of BP between outpatient visits based on telemonitoring and timely response to BP levels outside the target range. Thereby, the standard treatment group will profit as well due to greater awareness of their AH, as in every day practice AH is not treated sufficiently, with one third of KTx patients displaying BP values > 95th pct [8, 17].

Before and during the clinical trial, regular risk management will be performed according to good clinical practice (GCP). Every subject participating in the trial will be insured against any trial-related illness/injuries. An independent data safety monitoring board (DSMB) has been established, consisting of three independent experts, that oversees patient safety and assures the risk/benefit assessment while the trial is ongoing. Throughout the trial, the DSMB will monitor safety data according to a pre-specified plan (DSMB charter). DSMB members are independent from the PIs, the trial’s investigators, and the medical institutions involved. Together, the DSMB members form an independent multidisciplinary group of two clinicians and one biostatistician that, collectively, have experience in the management of KTx recipients and the conduct, monitoring, and statistical analysis of RCTs. The DSMB will give advice to PIs and the trial steering committee (TSC) whether to continue, modify, or stop the trial. Importantly, this will include the assessment whether the separation of BP values between the intensified and the standard group is sufficient. This will allow to take additional measures to reinforce BP goals early enough during the course of the trial. Detailed rules on the flow of data and communication between the parties involved in the trial and the DSMB have been set forth in a separate DSMB charter. The TSC also involves independent external experts that will review the trial’s recruitment and progress.

### Data security

An eCRF will be used by employing an electronic data capture system. The system has been fully validated according to the applicable criteria and regulations (e.g., GCP). Personal data of all participating patients will be handled in accordance with the General Data Protection Regulation (GDPR) of the European Union; all respective patient rights will be granted (Art. 12–23 GDPR). Pseudonymized data will be entered only by authorized and trained staff. Pseudonymization will be performed directly by the local investigator at the participating center. The local investigator has the only access to the pseudonymization key list and is therefore responsible for its safeguarding. A clinical trial data base allows data entries remotely via the eCRF. Data quality will be verified by centralized and repeated on-site monitoring by an independent, external CRO as well as via range, validity, and consistency checks programmed in the system. All changes of data entered in the eCRF will be documented by an audit trail. A quality control will be performed and documented before the database is closed. The complete data set will be archived in the central electronic MHH archive in compliance with the corresponding regulations. After data transfer, data analyses will be performed by the responsible biostatistician. The eCRF and the home BP telemonitoring data will be stored and processed on secure webservers within Germany during the duration of the study.

At the end of the study and after the final correction of the study database, the standard pseudonymization lists are archived in the site investigator file and must be kept for 10 years according to §13 Abs. 10 GCP-Regulation. The lists will then be destroyed. However, individual participants may declare their willingness to participate in follow-up surveys even after the end of the study and would thus be excluded from anonymization. Pseudonymized study data are archived for 25 years in accordance with IHC GCP E6 (R2).

Responsible for data management will be the principal investigator, Prof. Melk. The data protection officer at MHH is Joachim Barke.

### Biosampling

Collected biomaterials will be stored at Hannover Unified Biobank, which follows highest quality standards and is certified according to DIN EN ISO 9001:2015. The material will be used in context of the study-purpose only. The analyses of biochemical parameters will be performed in a centralized laboratory in Germany to guarantee comparability.

### Insurance

The subjects participating in the trial will be insured against any trial-related illness/injuries (insurance company: HDI Global SE).

## Discussion

The SOPHOCLES trial targets a central clinical problem highly relevant for pediatric KTx recipients and addresses a major knowledge gap. It aims to define new BP targets and thereby improve current BP management. Despite the strong association between AH and CV morbidity in this patient group, no specific BP targets have been established for children and adolescents after KTx. By comparing an intensified BP management strategy (target ≤ 60th pct) with standard BP management (target < 90th pct), this study seeks to determine whether intensified BP control leads to a significant reduction in LVMI, a known predictor of adverse CV outcomes. The results of this trial will have direct implications for post-KTx care, potentially reducing CV morbidity and improving overall patient outcomes.

In children not yet requiring KRT, the ESCAPE trial [24] demonstrated that intensified BP control by ACE inhibition slowed CKD progression. This RCT derived data changed BP management in the pediatric CKD population without KRT substantially. However, the results could not simply be applied to patients after pediatric KTx as this population is different in several aspects. Their graft generally provides them with good kidney function and does not suffer from progressive kidney disease. The often-occurring donor-recipient size mismatch results in changes in renal perfusion. Pediatric KTx recipients are rather exposed to classical CV risk factors including AH, but also dyslipidemia and diabetes due to immunosuppressive medication. Still, AH is in this cohort is the most prominent CV risk factor and of explicit importance as long-term outcome of children after KTx is mainly determined by CV events. Based on the differences described, progression of kidney disease as used in ESCAPE seemed not to a suitable endpoint for this patient cohort. RCTs with CV events or death as endpoints are not feasible in this population either as the number of events would be too small. LVM was chosen as it appears to be a valid surrogate parameter, not only is it a proven predictor of CV morbidity with a strong correlation to AH [15], but has also been shown to be responsive to improved BP management during the proposed duration of this trial [18, 25]. Support for our chosen approach and trial design comes from an analysis of observational data from the 4C-T study indicating that continuous exposure to BP levels well below the 75th was able to reduce LVMI meaningfully [25].

Despite being confident that our trial will show the superiority of more intensified BP lowering with regard to LVMI, equipoise is guaranteed, as the risk–benefit assessment will not solely rely on the assessment of the primary endpoint. Intensified BP management will probably increase the pill burden [47], which might facilitate non-adherence [48]. Side effects of antihypertensive treatments might occur. Some might even argue that high normal BP is needed in children to guarantee adequate graft perfusion due to a potential donor-recipient size mismatch. Hence, eGFR is a vital secondary endpoint in this trial.

The proposed trial utilizes an innovative telemonitoring approach, which on its own is an important step towards a more consequent employment of existing digital tools in patient care.

Taken together, the SOPHOCLES trial addresses a major gap of knowledge, is timely, uses a trial design with the most appropriate endpoints, and employs an innovative digital technology.

## Trial status

The current protocol version number is 1.2 as of December 5, 2024. Enrollment started mid December 2024 with the first patient being randomized on December 13, 2024. Recruitment period is 2 years and thus will be complete at the end of 2026. The current protocol adheres to the SPIRIT reporting guidelines [49]. The respective checklist can be found in the supplementary material.

## Supplementary Information

Additional file 1: SPIRIT checklist.

## Data Availability

We intend to publish the results of the trial. The publication rights are with the coordinating investigator. The coordinating investigator will assure that contributions of participating individuals and centers are acknowledged. The national study group headed by the physicians of the central clinical study office will have sole access to the final trial dataset. Anonymized clinical data including measurements of endpoints will be made available to other investigators after publication of the trial upon reasonable request for noncommercial use. This can be facilitated via the Hannover Medical School open access online repository.

## Abbreviations

AE: Adverse event
AH: Arterial hypertension
BP: Blood pressure
CERTAIN: Cooperative European Pediatric Renal Transplant Initiative
CV: Cardiovascular
DSMB: Data safety monitoring board
eGFR: Estimated glomerular filtration rate
GCP: Good clinical practice
GDPR: General Data Protection Regulation
IMT: Intima media thickness
KTx: Kidney transplantation
LVH: Ventricular hypertrophy
LVMI: Left ventricular mass index
MMRM: Mixed model for repeated measures
pct: Percentile
PWV: Pulse wave velocity
RCT: Randomized controlled trial
SAE: Serious adverse events
TSC: Trial steering committee
